# Avoiding contact allergens: from basic research to the in vitro identification of contact allergens 

**DOI:** 10.5414/ALX01440E

**Published:** 2017-08-04

**Authors:** S.F. Martin, P.R. Esser

**Affiliations:** Forschergruppe Allergologie, Hautklinik, Universitätsklinikum Freiburg, Germany

**Keywords:** skin, contact dermatitis, innate immune system, inflammation, in vitro assay

## Abstract

Allergic contact dermatitis (ACD) is a chemical-induced inflammatory skin disease. Contact allergens are low-molecular-weight chemicals that must react with proteins in order to become immunogenic. This interaction leads to the activation of innate immune and stress responses and to the formation of antigenic epitopes for T cells which are the effector cells of ACD. Due to the multitude of chemicals that surround us in our daily life and their potential sensitizing capacity, it is crucial to identify contact sensitizers before these chemicals are used in consumer products. Appropriate *in vitro* assays for hazard identification are urgently needed to replace animal-based assays. The EU-wide ban on sensitization testing of cosmetic ingredients in animals is in effect since March 2009 and the necessity to test more than 30,000 already marketed chemicals for their sensitizing potential under the EU regulation REACh has intensified the worldwide efforts to replace animal testing. We summarize here the current strategies to develop a battery of assays which allows the identification of contact allergens by *in vitro* alternatives to animal testing. Our main focus lies on the test systems recently developed within the EU project *Sens-it-iv* in which we participate.

German version published in Allergologie, Vol. 34, No. 11/2011, pp. 529-537

**Abbreviations:** ACD: allergic contact dermatitis; DC: dendritic cell; HA: hyaluronic acid; ICD,: irritant contact dermatitis (ICD); LC: Langerhans cell; NLR: NOD-like receptor; ROS: reactive oxygen species; TLR: Toll-like receptor

## Characteristics of contact allergens and strategies to avoid them 

**Figure 1.**
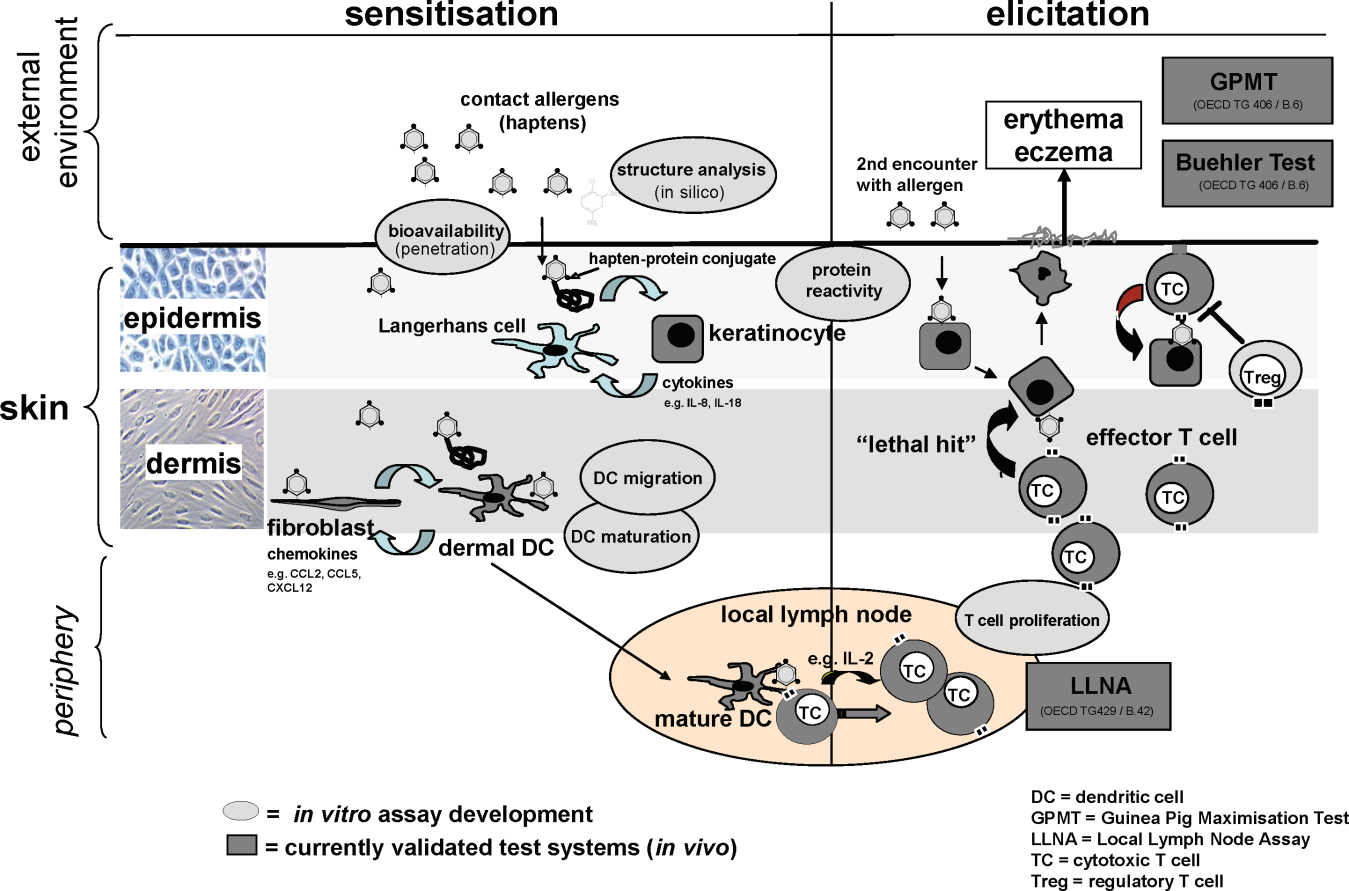
Steps leading to sensitization to contact allergens and *in vitro* assay development.**** *In vitro* assays for contact allergen identification must reproduce key steps of the sensitization phase of ACD. Skin penetration, protein modification and activation of innate immune and stress responses in the skin are followed by DC emigration and homing to skin draining lymph nodes. Contact allergens are presented on DC in the context of MHC molecules to T cells. These are primed and differentiate to effector T cells which exert their function after recruitment to the skin in the elicitation phase. The validated animal tests (GPMT, Buehler Test and LLNA) must be replaced by *in vitro* alternatives.

Allergic contact dermatitis (ACD) is among the most frequent skin diseases with increasing prevalence. It is very often work-related and clinically problematic due to the risk of chronification and the lack of causative treatments. Due to often long treatment times using immunosuppressive drugs such as corticosteroids which may have significant side effects the socioeconomic impact and the impact on the quality of life of the patients is enormous. Therefore, avoiding contact allergens is the optimal prevention and mandatory for patients. However, this is often not possible without a change of the profession when ACD is caused by contact allergens in the workplace. A reliable system for the identification of contact allergens by the chemical and pharmaceutical industry – so far done using animal-based assays – is essential to assure consumer safety [1, 2, 3, 4]. Mice and guinea pigs are used for sensitization testing. The only validated and accepted assays so far are the Guinea Pig Maximisation Test (GPMT) and the Buehler Test (OECD Test Guideline 406) as well as the mouse Local Lymph Node assay (LLNA). The LLNA and its modifications represent the current gold standard (OECD Test Guidelines 429, 442A, 442B) (Figure 1). In the original protocol test substances are applied daily for three consecutive days on mouse ear skin. Then, the proliferation of draining lymph node cells is assessed by *in vivo* incorporation of radioactive [^3^H]-thymidine. The so-called EC3 values quantify the stimulation over the control level and can be used to additionally assess the relative potency of the identified contact allergens. The latter is an important parameter due to the possibility to use substances with weak sensitizing potency at low concentrations in consumer products. Many fragrances used in perfumes, soaps and other products fall within this category. However, the EU legislation has banned sensitization testing in animals for the cosmetics industry since March 2009 with the exception of repeated dose testing until March 2013. In addition, the EU regulation REACh requires sensitization testing of more than 30.000, more likely 50 – 60.000 already marketed chemicals produced in amounts over 1 ton/year [5]. Therefore, and for financial and ethical reasons, *in vitro* alternatives to animal testing are being developed internationally. The EU has funded such a program, the project *Sens-it-iv* (Novel Testing Strategies for *in vitro* assessment of allergens, see also www.sens-it-iv.eu) from 2005 to 2011. The development of *in vitro *alternatives is a yearlong process including basic research. Establishing an *in vitro* assay with a standardized operation procedure (SOP) requires technology transfer and ring trials by independent laboratories with blinded chemicals for assay validation as well as regulatory acceptance for example by the European Centre for the Validation of Alternative Methods (ECVAM) and the responsible authorities in the EU and other countries. Examples for criteria that must be followed are given by the OECD Guidelines for the Testing of Chemicals (e.g. Guideline 34). 

**Figure 2.**
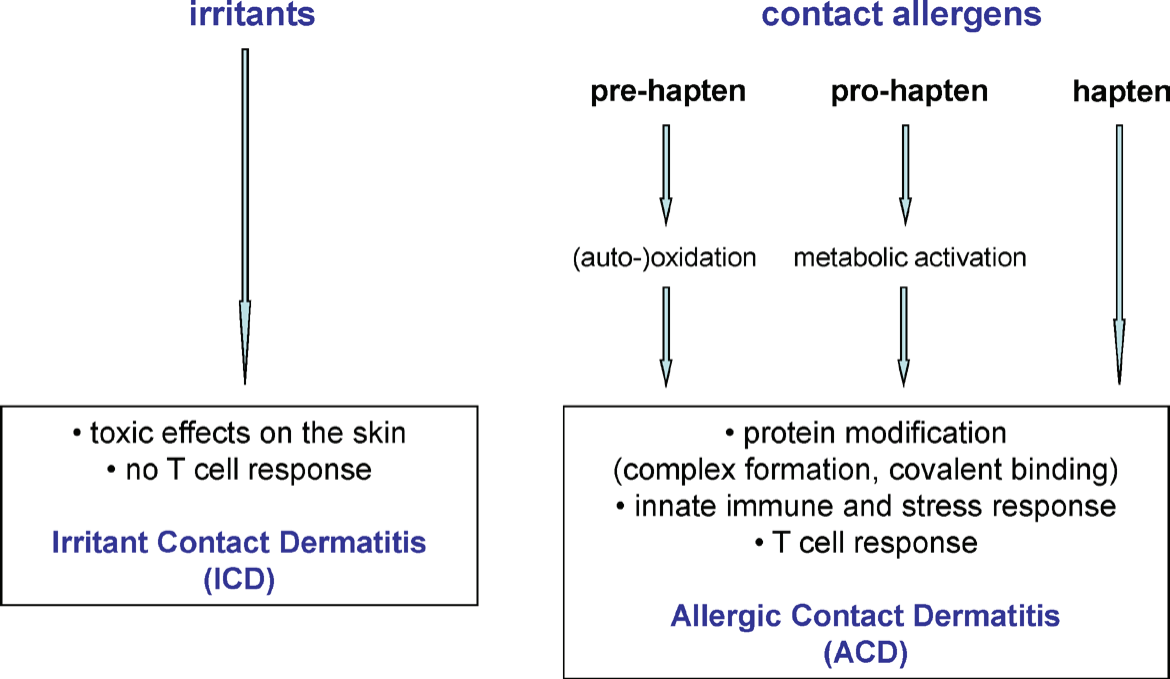
Currently used terminology for categories of chemicals that cause contact dermatitis. Irritants have toxic effects on the skin and induce Irritant Contact Dermatitis (ICD) without triggering a T cell response. In contrast, contact allergens are protein-reactive haptens or pre- and pro-haptens that are converted to reactive haptens by (auto-) oxidation or metabolic activation. They activate the innate immune system due to their adjuvant effect and form antigenic T cell epitopes leading to Allergic Contact Dermatitis (ACD).

More than 4.000 low molecular weight (< 500 Dalton) organic and inorganic chemicals are identified as contact allergens. They react with proteins either by complex formation, for example with histidines in the case of metal ions such as nickel and cobalt, or they covalently bind to amino acid side chains of e.g. cysteine and lysine as shown for many organic chemicals. In contrast to irritant chemicals which have toxic effects on the skin and induce irritant contact dermatitis (ICD) this chemical protein reactivity is a hallmark of contact allergens and a prerequisite for their immunogenicity and antigenicity. Therefore, they are called haptens or half-antigens. Protein reactivity is acquired by some chemicals, the so-called pre-haptens by (auto-) oxidation or for others, the so-called pro-haptens only after metabolic conversion (Figure 2). These pro- and pre-haptens are very problematic with respect to clinical diagnosis and *in vivo* assays for contact allergen identification. Usually, the parent compound that is used in clinical patch testing or in the *in vivo* assays lacks sensitizing potential and the reactive adducts are most of the time unknown. 

Parameters that are not fully addressed by the current *in vitro* assay strategies are the ability and efficiency of the chemical to penetrate into the skin and the frequency of application or skin contact that in some cases allows accumulation over a critical threshold for sensitization that is not reached by a single contact. Also the metabolic conversion and oxidation of pro- and pre-haptens is not yet well enough understood to be implicated in assays, but with the current progress in basic research such efforts are underway [6, 7, 8]. These problems must be solved in the future. 

## The sensitization phase of Allergic Contact Dermatitis 

The ultimate goal of all efforts to replace animal testing for contact allergen identification by *in vitro* assays is the implementation of key steps in the immune response to contact allergens in assay development (Figure 1). The unavoidable reductionism that is required for all *in vitro* assays must be able to reproduce key processes that happen in a given cell type in its tissue microenvironment *in vivo* during sensitization or elicitation of contact dermatitis. This is a great challenge and requires a detailed mechanistic understanding of the immunologic pathomechanisms of the disease. Especially in the field of innate immune responses to contact allergens significant progress has been made during recent years and has improved our understanding of the sensitization process [9, 10, 11]. One of the striking features of contact allergens besides their ability to form antigenic determinants for T cells is their adjuvant effect. This allows them to activate innate immune and stress responses which lead to skin inflammation. Most likely, some of the chemical protein modifications introduced by contact allergens act like conventional post-translational modifications by altering protein function, localization and by inducing signalling and, eventually, inflammation [10, 11, 12]. The inflammatory response is essential for the sensitization. Most likely it is also required for the elicitation of ACD for example to provide the cytokines and chemokines that are needed to recruit the contact allergen-specific effector T cells from the bloodstream to the inflamed skin. Studies in the mouse contact hypersensitivity (CHS) model have now shown that contact allergens trigger innate immune and stress responses that are also used in immune responses to infection [10, 11]. Thus, TLR2 and TLR4 play a role for CHS to TNCB, oxazolone and FITC [13]. These innate immune receptors recognize the bacterial cell wall components such as lipopeptides and lipopolysaccharide (LPS) from gram-positive and gram-negative bacteria, respectively. We have demonstrated that TNCB induces the degradation of the extracellular matrix component hyaluronic acid (HA). Fragments of HA then trigger TLR2 and TLR4 to promote skin inflammation (Esser, PR. In preparation, Abstract P095, Allergologie 20:177, 2011). In contrast, nickel ions interact directly with conserved histidines in the human TLR4 and induce signalling [14]. Interestingly, these histidines are missing in the mouse TLR4 which explains why mice could not be used as an animal model for ACD to nickel. In order to induce CHS in mice, nickel must be co-applied with adjuvants such as complete Freund’s adjuvant or LPS [15, 16, 17]. These results highlight the crucial role of the adjuvant effect of contact allergens and the essential role of innate immunity in ACD. 

Contact allergens also induce the production of reactive oxygen species (ROS) which participate in the HA degradation and activate Keap1/Nrf2 mediated anti-oxidant responses. They can also deplete glutathione thereby causing a disturbed redox balance of the cells [18, 19, 20, 21, 22, 23, 24]. 

Another action of contact allergens is the triggering of ATP release from stressed or damaged cells in the skin [25]. Extracellular ATP serves as a danger signal and triggers an efflux of K^+^ ions from the cells via stimulation of the purinergic ATP receptor P2X_7_. This, in turn leads to the activation of the NLRP3 inflammasome, a cytosolic protein complex that activates caspase-1. This enzyme then cleaves the immature pro-forms of IL-1 and IL-18 which are produced in response to TLR stimulation to the mature and secreted forms. These cytokines are important mediators of inflammation, and IL-1R signalling is essential for sensitization to contact allergens. The cooperative action of these innate immune and stress pathways is crucial in DC [10, 11]. Failure of a single one (TLR2/4 activation, ROS production, P2X_7_ triggering, NLRP3 inflammasome activation) is sufficient to abrogate the sensitizing potential of DC [13, 14, 15, 16, 17, 18, 19, 20, 21, 22, 23, 24, 25]. These findings form a scientific and conceptual framework for the development of new *in vitro* assays or the improvement of existing assays. Further progress in assay development will be made by implementing the results from genomic and proteomic studies. These global techniques aim at identifying profiles of genes or proteins that are regulated in their expression by contact allergens but not by irritants. In addition, such contact allergen-specific signatures help to confirm known and to identify new signalling pathways involved in the sensitization process. This will also be useful for the identification of new drug targets for causative treatment of ACD. 

## In vitro assay development for contact allergen identification 

*In vitro* assays for contact allergen identification must cover the key steps of the sensitization process (Figure 1). Several of the *in vitro* assays that have been and are being developed address the interaction of chemicals with proteins or cells such as monocytes, DC [26] and keratinocytes [27, 28]. One of the first steps of the sensitization phase is the protein binding of contact allergens based on their essential chemical reactivity. Understanding the chemistry of contact allergens is one of the goals that may allow *in silico* prediction based on structural alerts [29, 30]. Currently, covalent binding of chemicals to amino acid side chains is addressed by the Direct Peptide Reactivity Assay (DPRA) [31, 32]. The depletion of chemically modified model peptides with defined target amino acids for contact allergen modification is measured. This assay may also allow potency assessment since the efficiency of depletion that reflects the chemical reactivity correlates well with the EC3 values from the LLNA [33, 34]. The next step is the activation of skin cells. The human Cell Line Activation Test (hCLAT) uses the human monocytic leukemia cell line THP-1 which is exposed for 24 h to the test chemicals. Readout parameters are the induction of the co-stimulatory molecules CD54 and CD86 on the surface of these cells [35, 36, 37, 38]. Similarly, the Myeloid U937 Skin Sensitisation Test (MUSST) detects the induction of IL-8 and CD86 [39, 40]. Similar readouts as well as p38 MAP kinase activation have been used for THP-1 cells [41]. 

Epidermal keratinocytes and dermal fibroblasts also respond to contact allergens and irritants and significantly contribute to ACD and ICD. They produce inflammatory mediators such as cytokines and chemokines that attract immune cells into the skin and regulate the emigration of DC to the skin draining lymph nodes. Thus, human keratinocytes are triggered via TLR to produce pro-IL-18 and process it via the NLRP3 inflammasome to the bioactive mature cytokine. The NCTC assay uses the human keratinocyte cell line NCTC 2544 to assess intracellular production of IL-18 as triggered in response to contact allergens [27, 28]. 

DC migration from the epidermis to the dermis and then to the draining lymph nodes is the next step in sensitization that can be addressed by *in vitro* assays. It has recently been shown that both irritants and contact allergens induce the migration of Langerhans cells (LC) from the epidermis to the dermis. This step precedes the CCR7 and CCL19/CCL21 dependent migration via the efferent lymphatics to the draining lymph node where the priming of contact allergen-specific T cells occurs. It is dependent on a set of chemokines produced by dermal fibroblasts in response to both classes of chemicals [42, 43, 44]. The DC migration assay is based on the finding that irritants and contact allergens induce different chemokine receptors on Langerhans cells of the epidermis [45]. Thus, irritants induce CCR2 and CCR5 and, hence, LC migration to CCL2 and CCL5, while contact allergens induce CXCR4 and migration to CXCL12. The DC migration assay uses the human acute myeloid leukemia cell line MUTZ-3 which is differentiated to a LC-like phenotype [45]. The MUTZ-LC are then treated with the test chemical for 24 h. Subsequently they are used in a transwell migration assay using CCL5 or CXCL12 as chemoattractants. The migration index calculated as the CXCL12:CCL5 ratio serves as readout. The DC migration assay is up to now the only functional single cell test for contact allergen identification. 

A two-tiered strategy was proposed to assess the potency of contact allergens. The NCTC 2544 assay is used for contact allergen identification in the first tier [27, 28] and the EE potency assay for potency assessment in the second tier [46]. The latter assay addresses chemical toxicity and IL-1 production in epidermal equivalents as readout. The effective chemical concentration required to reduce cell viability by 50% (EE-EC50) and the chemical concentration which increases IL-1α secretion by 10-fold (EE-IL-1α[10×]) are used to determine sensitizer potency. 

A promising strategy to identify at least cysteine-reactive contact allergens uses a keratinocyte-based reporter cell line that expresses luciferase under the control of tandem anti-oxidant response elements (ARE) [23]. Contact allergens can react with cysteines in the cytosolic protein Keap1, a sensor for oxidative and electrophilic stress. This protein is associated with the transcription factor Nrf2. Nrf2 is usually fed to the proteasome for degradation but Keap1 oxidation or chemical modification at critical cysteines results in translocation of Nrf2 to the nucleus where it binds to ARE in genes of the anti-oxidant phase 2 response such as glutathione reductase, heme oxygenase and NAD(P)H:quinone oxidoreductase 1 [47, 48, 49]. The HaCaT human keratinocyte reporter cell line is now used to identify cysteine-reactive contact allergens based on their ability to trigger ARE-dependent luciferase activity [23]. 

Genomic studies have been performed with MUTZ-3 progenitor cells and monocytes that were treated with test chemicals. Characteristic gene profiles have been identified that can be used as a contact allergen-specific signature in the Genomic Analysis Rapid Detection (GARD) assay (www.sens-it-iv.eu and [50]). Similar approaches are ongoing in proteomic studies to identify characteristic protein signatures in MUTZ-3 and keratinocytes. Further refinement of these promising genomic and proteomic assays will clarify whether they could be stand-alone assays that reliably identify contact allergens without integration in a tiered strategy and whether or not potency assessment will be possible using these approaches. 

## T cell responses to contact allergens and assay development 

The most specific response of the immune system to contact allergens is the T cell response that concludes the sensitization phase. Contact allergens form T cell epitopes by chemical modification of peptides presented in the binding groove of MHC molecules on DC and other cells. Alternatively they can modify extracellular or cellular proteins which are then processed by DC to yield hapten-modified peptides for presentation to T cells after DC migration from the skin to the draining lymph nodes. Naïve T cells can recognize these haptenated peptides or complexes of metal ions with MHC molecules, peptides and TCR and become activated [51]. This T cell priming step leads to the proliferation of contact allergen-specific T cells and their differentiation to effector cells which are recruited to the inflamed skin during the contact allergen-induced inflammatory response in the elicitation phase of ACD. Based on these processes *in vitro* T cell priming assays (TCPA) have been developed [51]. The latest improved protocols for these functional tests use monocyte-derived DC as antigen-presenting cells (APC) and naïve human T cells. The sorting of naïve T cells or the depletion of CD25+ and CD56+ T cells may increase the sensitivity of the assay due to the depletion of immunoregulatory cells such as CD4+CD25+FOXP3+ regulatory T cells and NKT cells [52, 53]. The test chemical can be directly added to the co-cultures, DC can be pulsed before co-culture with T cells or haptenated proteins such as contact allergen-coupled human serum albumin can be used as antigens [51, 53]. The readout of the TCPA is T cell proliferation or, more efficient and specific, multiparametric flow cytometry to detect intracellular cytokines such as IFN- on defined populations of T cells [51]. The latter technique may allow potency assessment since the frequency of antigen-specific T cells is determined as well. We are currently testing whether the frequency of contact allergen-specific T cells and the diversity of their TCR repertoire correlates with the potency of the respective contact allergen. If this was the case such studies would allow assessment of contact allergen potency in the TCPA (Kimber, I. et al., submitted for publication) [39]. 

## Summary 

The current worldwide efforts to replace animal testing in the sensitization testing of chemicals is fuelled by the progress in basic research. The improved understanding of cellular and molecular processes in the sensitization and elicitation of ACD can now be implemented in assay development. The assays in development as well as future assays can be based on this knowledge. A tiered strategy is the likely future of *in vitro *assay development for the identification of contact allergens [54]. Ideally a battery of assays will be used that cover different steps of the sensitization process. A scoring system will then allow the identification of contact allergens and potency assessment. The fruitful international cooperations should also help to reduce animal testing not only for cosmetic ingredients but also in other areas of (immuno-) toxicology and research, to refine existing assays, and to eventually replace it [1]. 

## Acknowledgements 

The work reviewed here was supported in part by a grant of the European Commission as part of the project “Novel Testing Strategies for In Vitro Assessment of Allergens (Sens-it-iv)”, LSHB-CT-2005-018681.
